# Antimicrobial and Anti-Inflammatory Activities of Endophytic Fungi *Talaromyces wortmannii* Extracts against Acne-Inducing Bacteria

**DOI:** 10.1371/journal.pone.0097929

**Published:** 2014-06-02

**Authors:** Alexander Pretsch, Michael Nagl, Katja Schwendinger, Birgit Kreiseder, Martina Wiederstein, Dagmar Pretsch, Miroslav Genov, Ralph Hollaus, Daniela Zinssmeister, Abdesamad Debbab, Harald Hundsberger, Andreas Eger, Peter Proksch, Christoph Wiesner

**Affiliations:** 1 SeaLife Pharma GmbH, Tulln, Austria; 2 Medical and Pharmaceutical Biotechnology, University of Applied Sciences, Krems, Austria; 3 Institute of Pharmaceutical Biology and Biotechnology, Heinrich-Heine University, Düsseldorf, Germany; University of Leuven, Rega Institute, Belgium

## Abstract

Acne vulgaris is the most common skin disease, causing significant psychosocial problems such as anxiety and depression similar to a chronic illness for those afflicted. Currently, obtainable agents for acne treatment have limited use. Thus, development of novel agents to treat this disease is a high medical need. The anaerobic bacterium *Propionibacterium acnes* has been implicated in the inflammatory phase of acne vulgaris by activating pro-inflammatory mediators such as the interleukin-8 (IL-8) via the NF-κB and MAPK pathways. *Talaromyces wortmannii* is an endophytic fungus, which is known to produce high bioactive natural compounds. We hypothesize that compound C but also the crude extract from *T. wortmannii* may possess both antibacterial activity especially against *P. acnes* and also anti-inflammatory properties by inhibiting TNF-α-induced ICAM-1 expression and *P. acnes*-induced IL-8 release. Treatment of keratinocytes (HaCaT) with *P. acnes* significantly increased NF-κB and activator protein-1 (AP-1) activation, as well as IL-8 release. Compound C inhibited *P. acnes*-mediated activation of NF-κB and AP-1 by inhibiting IκB degradation and the phosphorylation of ERK and JNK MAP kinases, and IL-8 release in a dose-dependent manner. Based on these results, compound C has effective antimicrobial activity against *P. acnes* and anti-inflammatory activity, and we suggest that this substance or the crude extract are alternative treatments for antibiotic/anti-inflammatory therapy for acne vulgaris.

## Introduction

Acne vulgaris is a multifactorial disorder of the skin, affecting all ages and ethnic groups but usually occurs in teenagers [1]. In Caucasian populations, almost 85% of young adults between 12 and 25 years suffer from acne. Of this group between 15–30% display moderate to severe acne symptoms requiring medical treatment [2]. The pathogenesis of acne is multifarious, but the development of the disease involves increased sebum production by the influence of androgens, abnormal ductal keratinization resulting in comedons, bacterial hyper colonization and inflammation [3], [4]. Increased sebum secretion is associated with the development of acne lesions, since sebum serves as a nutrient source for the Gram-positive bacterium *Propionibacterium acnes* (*P. acnes),* a member of the normal skin flora [5]. Microbiological colonization of the sebaceous gland has been identified as a major factor in disrupting the follicular epithelium, in which *P. acnes* produce enzymes such as lipases, proteases, and hyaluronidases leading to subsequent inflammatory reactions in the surrounding dermis [6], [7]. In addition to its ability to stimulate C5a formation, *P. acnes* also secretes chemotactic factors for leukocytes which subsequently infiltrate in the hair follicle resulting in the destruction of the hair follicle wall [8]. This bacterium is thought to further contribute to inflammation through activation of the toll-like receptors TLR2 and TLR4, expressed on keratinocytes, sebocytes, and monocytes, leading to release of proinflammatory cytokines/chemokines such as interleukin1α, IL-6, IL-8, tumor necrosis factor (TNF)-α, and adhesion molecules like intercellular adhesion molecule-1 (ICAM-1) [9], [10]. IL-8 is a strong chemotactic factor for neutrophils, basophils, and lymphocytes [11] and is produced by keratinocytes in response to *P. acnes* treatment [12]. This process is mediated by the activation of the transcription factor nuclear factor-kappa B (NF-κB) and mitogen-activated phosphokinase (MAPK) pathway [9], [10], [13].

The therapeutic agents available for the treatment of acne (both oral and topical) targets different physiological aspects since they are employed to kill bacteria, prevent inflammation or comedo production [14], [15]. Even the most effective treatments require several months until they show noticeable effects and they are also known to induce side effects [16]. Therefore, the development of novel agents for acne, that have no side effects, but high antibacterial and anti-inflammatory activity are of particular importance.

The endophytic fungus *Talaromyces wortmannii* isolated from *Aloe vera* (Asphodeloideae) grown in Egypt has been proved to show high antimicrobial activity. Bara et al. (2013) clearly demonstrated that all six compounds isolated from *T. wortmannii* exhibited antibacterial activity, predominantly directed against *Staphylococcus aureus*, including even high-level (multi)drug-resistant isolates whereas other Gram-positive genera such as *Streptococcus*, *Enterococcus* or *Bacillus* were only moderately affected [17].

Based on the previously known antibacterial effects of *T. wortmannii*, we isolated different compounds (A, A, B, B, C and D) from *T. wortmannii* that were subsequently proved to be members of the flavomannin family of compounds and investigated their potential to be effective acne treatments. In this manuscript, we demonstrate specifically the antibacterial potential of compound C and the crude extracts as having high antibacterial activity against *P. acnes* and *S. epidermidis* but also reporting their low toxicity to skin cells. Moreover, compound C was found to prominently down-regulate ICAM-1 expression in endothelial cells after TNF-α stimulation and IL-8 secretion in keratinocytes after *P. acnes* treatment via the NF-κB and MAPK pathways. Therefore, according to these results, we suggest that compound C from *T. wortmannii*, but also the crude extract may be employed as effective therapeutic agents to improve acne disease.

## Materials and Methods

### Fungal Material and Cultivation

The endophytic fungus *Talaromyces wortmannii* was kindly provided by Prof. Peter Proksch [18]. Mass growth of the fungus for the isolation and identification of new metabolites was carried out in Erlenmeyer flasks (1 L each). The fungal strain was cultivated on rice solid medium (100 mL of distilled water was added to 100 g commercially available rice and kept overnight prior to autoclaving) for 14–21 days (until the rice turns green to yellow) at 22°C under static conditions.

### Extraction and purification of fungal secondary metabolites

Cultures were cut into little pieces and extracted with EtOAc overnight during shaking. After centrifugation and filtering, organic phase was washed with demineralized water, the solvent phase evaporated and the residue dried under high vacuum overnight. Crude extract was subjected to chromatographic separation via normal phase flash chromatography on Silica gel 60 (0.040–0.063 mm) utilizing a solvent step gradient (DCM/MeOH 9∶1 to 2∶1). Fractions were analytically screened via HPLC C8 column equilibrated with 85% ddH_2_O, 1% HCOOH and 15% ACN. The extract was eluted with a solvent gradient (ACN-0.1% aq HCOOH to 100% ACN) at a flow rate of 0.3 ml/min.

To isolate the different components A-C, the obtained Flash Chromatography fractions were subjected to Size Exclusion Chromatography consisting of a Sephadex-gel (LH-20) as stationary phase and a solvent mixture of CHCl_3_:MeOH:Cyclohexan (2∶2∶1) as mobile phase. Component D was obtained directly after Flash Chromatography, compound C after Flash and Sephadex LH-20 Chromatography from fraction 3, compounds B and B from fraction 4 and compounds A and A from fraction 5.

### Bacterial culture

Propionibacterium acnes, Staphylococcus epidermidis, Streptococcus pneumoniae, Enterococcus faecalis, MRSA (MET^R^), E. coli and Klebsiella pneumoniae belong to the SeaLife Pharma MDR pathogen collection and were isolated from infected patients. S. epidermidis, S. pneumoniae, E. faecalis, MRSA (MET^R^), E. coli and K. pneumoniae were cultivated in Mueller Hinton Broth liquid (Carl Roth, Karlsruhe, Germany). Propionibacterium acnes_5–7 were obtained from the Department of Internal Medicine I, Division of Infectious Diseases and Tropical Medicine, Medical University of Vienna. P. acnes strains were cultivated under anaerobic conditions (GENbag, bioMerieux) in reinforced clostridium medium (Oxford, Hampshire, England) and Brucella agar plates (Sigma Aldrich) at 37°C. Erythromycin-resistant mutants were obtained by serially passaging the P. acnes strain in 96 wells containing a maximum of 25 µg/L Erythromycin in agar, as described previously [35]. For keratinocyte stimulation, P. acnes strains were grown for 5 days (stationary phase) and harvested by centrifugation at 2,000 g for 15 min at 4°C. Pellets were washed twice with cold PBS and suspended in PBS at approximately 10^7^–10^9 ^CFU/ml. Bacteria were heat-killed at 60°C for 20 min and 5 µl of heat-killed bacteria cultures were added in each well (100 µl) to stimulate the keratinocytes.

### Determination of minimal inhibitory concentration

Tests were carried out according to the EUCAST (http://www.eucast.org) criteria in a dilution assay with compound concentrations between 250 and 0.48 µg/ml. The minimal inhibitory concentration (MIC) of a substance was defined as the lowest concentration where bacterial growth was inhibited. *P. acnes* strains were cultivated under anaerobic conditions (GENbag, bioMerieux) at 37°C.

### Biochemicals and antibodies

Purified nonlabeled monoclonal mouse and rabbit antibodies were anti-phospho-ERK1/2, ERK1/2, anti-phospho-JNK/SAPK, anti-JNK/SAPK, anti-phospho-IkappaB-alpha (Cell Signalling Technology), monoclonal anti-human ICAM-1 (CD54) purified mouse Immunoglobulin and isotype-matched control IgG (Sigma, Austria), secondary goat anti-mouse antibody-Alexa 488 (Sigma, Austria), HRP-conjugated polyclonal anti-tubulin polyclonal antibody (Abnova). TNF-α and INF-γ were purchased from PeproTech (Rocky Hill, NJ). 5 x NF-kappaB and AP-1 Reporter were purchased from Stratagene.

### Cells and cell cultures

HEK 293 cells, primary keratinocytes (HKER), primary umbilical vein endothelial cells (HUVECs), CaCo-2 cells and HeLa cells were obtained from ATCC, and human adult low calcium high temperature keratinocytes (HaCaT) cells were obtained from DKFZ (Germany).

### Cell Viability Assay

Cells (HeLa, CaCo-2, HKER, HaCaT and HUVEC) were plated at 2×10^5^ cells/ml in 96 well plates (100 µl/well) for 6 h, prior to stimulating them with different concentrations (serial dilution starting with 500 µg/ml) crude extract or compound AA, BB, C and D (serial dilution starting with 250 µg/ml) or left untreated (neg. contr.) for 24 h. Subsequently, cells were incubated for 3 h with 10 vol% of Cell Titer Blue (Promega), and cell viability was assessed at an excitation wavelength of 530 nm and an emission wavelength of 590 nm in the multiplate reader, Tecan Infinite 200pro.

### Western blotting

For Western blotting, proteins were extracted from 10^5^ HaCaT cells. Total protein extracts were separated by SDS PAGE (4%–20%, BioRad) and transferred to Hybond C nitrocellulose membranes (BioRad). Membranes were blocked with 5% nonfat milk in Tris-buffered saline (pH 7.4), and immunodetection was carried out using specific antibodies (see ‘‘Biochemicals and antibodies’’ section).

### Cell transfection and reporter assay

HaCaT cells (10^5^ per well) were transiently transfected by the lipofectamin-2000 method using 2 µg DNA (Reporter plasmid) and 7 µl lipofectamin (Invitrogen). After 6 h, transfected cells were rinsed and incubated for additional 16 h before they were plated on 96 well plates. 24 h later HaCaT cells were pre-treated with compound C, crude extract or left untreated for 1 h and inflammation stimulated with heat-killed *P. acnes* suspension (see bacterial culture) or 10 ng/ml TNF-α for further 8 h. Cells were lysed and luciferase measured using a multiplate reader (Tecan Infinite 200).

### Flow cytometric analysis

For FACScan, cells (2–4×10^5^) were harvested, resuspended in PBS and 0.1% BSA and stained with AnnexinV/ PropidiumIodid (Biological Industries) for 1 h on ice. After staining, cells were analyzed using flow cytometry and software (Accuri).

### Electrical cell-substrate impedance sensing technology

HUVEC monolayer rearrangement and toxicity assay were performed with the ‘‘Electrical Cell-substrate Impedance Sensing’’ (ECIS) model 9600Z (Applied BioPhysics). The measurement system consists of a 96-well cell culture dish (96W10E plate) with ten active electrodes. 1×10^5^ HUVEC cells were grown to confluent monolayers and treated with different concentrations of the crude extract from *T. wortmannii*. Cytotoxicity and monolayer breakdown was assessed by continuous resistance measurements for 24 h.

### Cell-based ELISA for ICAM-1 Expression

ICAM-1 expression assay was performed as described previously [19]. Shortly, HUVECs (2×10^4^/ml), grown in either transparent 96-well plates and adhered for 6 h, were incubated for 1h with 25 µg/ml compound AA, BB, C, D or crude extract and stimulated with the pro-inflammatory cytokine TNF-alpha (20 ng/ml) for further 18 h. Subsequently, the level of ICAM-1 expression can be quantified by means of cell-based ELISA. Upon washing with PBS and fixing the cells for 2 min with methanol (−20°C), wells were subsequently blocked from unspecific binding with PBS+5% BSA. Subsequently, ICAM-1 expression was assessed using a monoclonal anti-human ICAM-1 (CD 54) (Clone 8.4A6) purified mouse Immunoglobulin (1 µg/ml, Sigma, Austria) or an isotype-matched control IgG (1 µg/ml, Sigma, Austria) as the primary antibody and a goat anti-mouse polyclonal antibody labelled with Alexa-488 (1 µg/ml, Sigma, Austria) as a secondary antibody. After washing, the fluorescence was then detected in the multiplate Reader (EX/EM 485/535). The % of ICAM-1 upregulation was then calculated as follows: % of basal ICAM-1  =  (Test Signal –Control antibody signal)/(Basal signal – control antibody signal) x 100%.

### Detection of IL-8

HaCaT cells (3×10^4^ per well, in 96well plates) were treated with compound AA, BB, C, D or with the crude extract or different concentrations of compound C and stimulated with *P. acnes* suspensions (see bacterial culture) after 2 h (pre-treatment), at the same time (co-treatment) or 3 h before (post-treatment). After 24 h Interleukin-8 was detected using the Human IL-8 ELISA kit (Life Technology, USA) according to the manufacturer's instructions.

### Statistical analysis

The Student's paired t-test was used for analysis. Reported p values are three-tailed; *, p<0.05 was considered statistically significant, and **, p<0.01 were considered as statistically highly significant.

## Results

### Isolation of novel compounds from *Talaromyces wortmannii* extract

**Figure 1:**
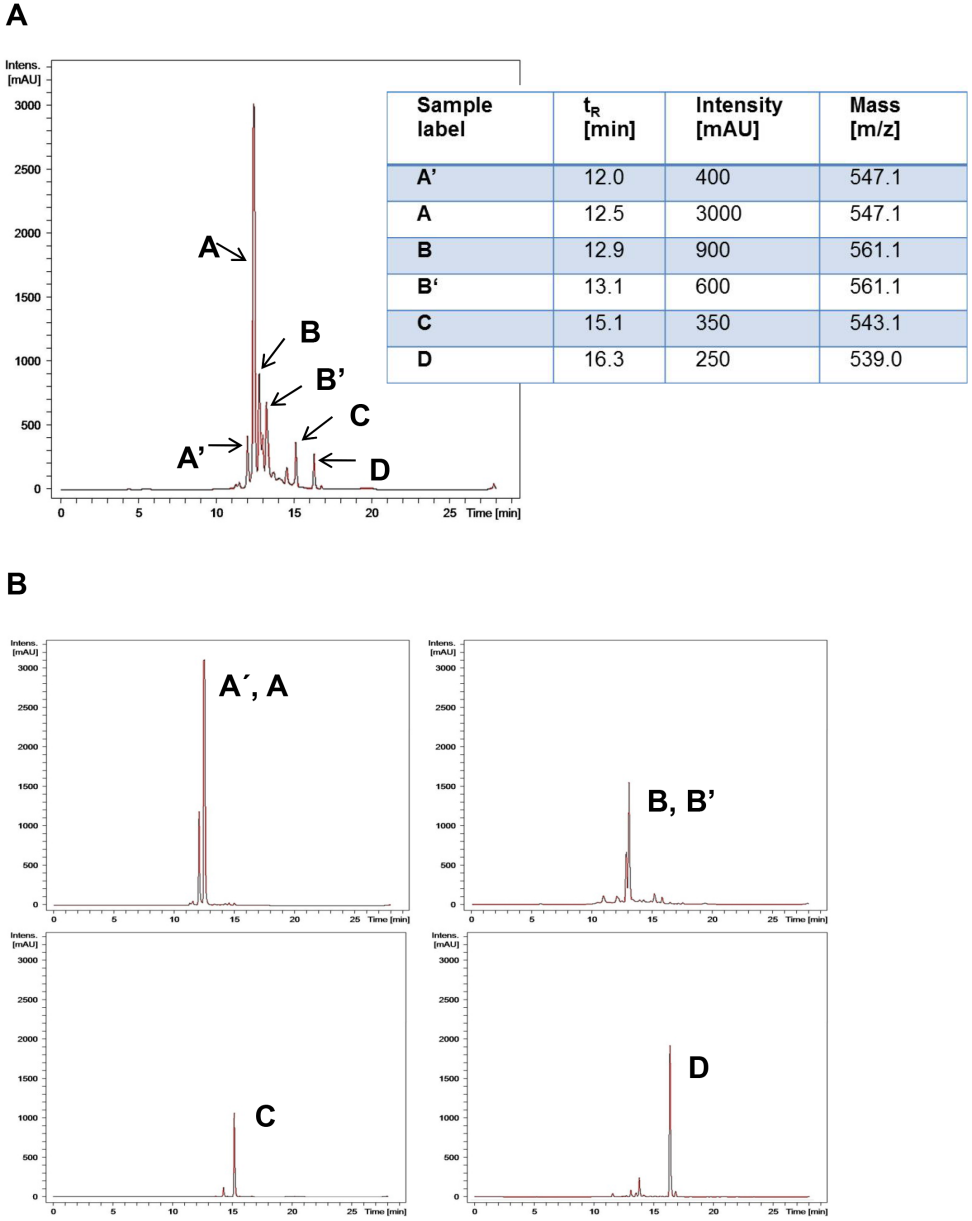
Analytical HPLC/MS profiles. (A) HPLC/MS profile of the crude extract of *T. wortmannii* isolated with EtOAc. (B) Chromatogram after straight phase and sephadex-G50 separation.

Cultures from *Talaromyces wortmannii* were extracted with EtOAc and separated chromatographically via silica gel flash chromatography into 8 crude fractions. These crude fractions were then analysed using HPLC as described in the relevant section within Materials and Methods. Figure 1 A displays a set of six main compounds assigned as AA, BB, C and D. Peak A indicated a retention time of 12.0 min and an intensity of 400 milli absorption units (mAU), A of 12.5 min and 3000 mAU, B of 12.9 min and 900 mAU, B of 13.1 min and 600 mAU, C of 15.1 min and 350 mAU and D of 16.3 min and 250 mAU (Fig. 1 A). ESi and MS m/z values for the individual compounds were obtained as: m/z 547.1 [M+H]^+^ (A, A), m/z 561.1 [M+H]^+^ (B, B), m/z 543.1 [M+H]^+^ (C) and m/z 539.0 [M+H]^+^ (D). For compound isolation, the crude fractions, obtained by flash chromatography, were subjected to size exclusion chromatography (Sephadex) as described in Material and Methods. Fig. 1 B shows HPLC/MS profiles of the isolated extract components AA, BB, C and D.

### Antibacterial assays used to test compounds and extracts of *Talaromyces wortmannii*

To elucidate the antibacterial activity of *Talaromyces wortmannii* extract against Gram-positive and Gram-negative bacteria, the minimum inhibitory concentration (MIC), which is the lowest concentration yielding no growth were determined (Table 1). Crude extract preparations of *T. wortmannii* were found to have significant antibacterial activities against Gram-positive bacteria followed by moderate activity against gram-negative bacteria. As shown in Table 1, *T. wortmannii* inhibited the growth of Gram-negative bacteria within a range of 62.5–125 µg/ml and Gram-positive bacteria within a range of 3.9–31.5 µg/ml. The best anti-bacterial activity of *T. wortmannii* was indicated against *Propionibacterium acnes* (3.9 µg/ml) followed by *Staphylococcus epidermidis* (7.8 µg/ml), *Enterococcus faecalis* (7.8 µg/ml) and *MRSA* (15.7 µg/ml). The antibacterial activity of the pure compounds against *P. acnes*, *S. epidermidis* and *MRSA* were found to be significantly improved than that obtained for the crude extract (see table 2).

**Table 1:** Minimal inhibitory concentrations (MIC in µg/mL) of the crude extract isolated from *T. wortmannii* including resistance profile of the tested pathogens.

Gram +	Resistance	Extract µg/ml	Gram -	Resistance	Extract µg/ml
***Propionibacterium acnes***	susceptible	3.9	***Escherichia coli***	susceptible	125
***Staphylococcus epidermidis***	BAC^R^	7.8	***Klebsiella pneumoniae***	KAN^R^	62.5
***Streptococcus pneumoniae ATCC 49619***	susceptible	31.5	***Pseudomonas aeruginosa***	CAZ^R^, CIP^R^, MER^R^,GEN^R^, IMI^R^, MER^R^, PIP/TAZ^R^	125
***Enterococcus faecalis***	CLA^R^, ERY^R^, MXF^R^, TEL^R^, VRE	7.8	***Salmonella enteritidis***	susceptible	62.5
***MRSA***	MET^R^	15.7			

Crude extract was tested in a 96 well dilution assay according to the EUCAST criteria and overlaid with bacteria solution in a concentration of 10^4^ bacteria/ml. After 24 h the minimal inhibitory concentration (MIC) of a substance was defined as the lowest concentration where bacterial growth was inhibited. Tests were done in triplicates and the table shows the average value of multiple MIC tests.

Antibiotic abbreviations for resistance: *MRSA*: *Methicillin-resistant Staphylococcus aureus*, *VRE*: *vancomycin-resistant enterococci*; CAZ (ceftazidime), CIP (ciprofloxacin), CLA (clarithromycin) DOX (doxycycline), ERY (erythromycin), GEN (gentamycin), KAN (kanamycin), MER (meropenem), MET (methicillin), BAC (bactroban), MXF (moxifloxacin), PIP/TAZ (piperacillin/tazobactam),TEL (telithromycin).

**Table 2:** Minimal inhibitory concentrations (MIC in µg/ml) of the isolates AA, BB, C, D and control.

	*Talaromyces wortmannii* components (µg/ml)				Control (µg/ml)		
***Isolates***	**AA‘**	**BB‘**	**C**	**D**	**Chloramphenicol**	**Ampicillin**	**Vancomycin**
***Propionibacterium acnes***	62.5	15.7	0.98	7.8	0.98	0.98	1.98
***Staphylococcus epidermidis***	31.25	7.81	0.24	3.91	3.91	3.91	3.91
***MRSA***	62.5	31.2	1.9	3.9	3.91	3.91	3.91

Isolates were tested against skin pathogens like *MRSA*, *Propionibacterium acnes* and *Staphylococcus epidermidis* in a classical MIC dilution assay to define the lowest concentration for *in vitro* inhibition. Therefore, the compounds or fractions were diluted between 250 and 0.48 µg/ml and overlaid with bacterial solution (10^4^ bacteria/ml). The table indicates that the isolated compound C shows the best activity against all tested bacteria.

As listed in Table 2, BB, C and D showed strong antimicrobial abilities against *P. acnes*, *S. epidermidis* and *MRSA*. The MICs values of BB, C and D against *P. acnes* were 15.7 µg/ml, 0.98 µg/ml and 7.8 µg/ml, respectively. The component BB, C and D showed also strong antimicrobial functions against *S. epidermidis* (MIC of 7.8 µg/ml, 0.24 µg/ml and 3.91 µg/ml) and *MRSA* (MIC of 31.2 µg/ml, 1.9 µg/ml and 3.9 µg/ml), whereas component A indicated antibacterial activity only in high concentrations (31.5–62.5 µg/ml). In comparison to commercially available antibiotics (control) such as chloramphenicol, ampicillin and vancomycin, compound C displayed an equally good or even better antibacterial activity against *P. acnes*, *S. epidermidis* and *MRSA* (Table 2). Furthermore, compound C showed strong antibacterial activity against all tested *P. acnes* strains (MIC of 0.49–1.98 µg/ml) and an antibacterial activity comparable to minocycline (0.25–3.91 µg/ml), erythromycin (0.98–1.98 µg/ml) and doxycycline (0.49–1.98 µg/ml), the most frequently used antibiotics to treat acne vulgaris (Table 3).

**Table 3:** Minimal inhibitory concentrations (MIC in µg/mL) of the *T. wortmannii* compound C in comparison with antibiotics used for acne treatment on different *P. acnes* strains.

P. acnes strains	Compound (µg/ml)	Control (µg/ml)		
	**C**	**Minocycline**	**Erythromycin**	**Doxycycline**
**P. acnes**	0.98	0.25	0.98	0.49
**P. acnes (ERYR)**	0.98	0.25	>250	0.49
**P. acnes_5**	1.98	3.91	1.98	1.98
**P. acnes_6**	0.49	1.98	1.98	0.98
**P. acnes_7**	1.98	7.81	0.98	0.98

Compound C, minocycline, erythromycin and doxycycline were tested in a 96 well dilution assay under anaerobic conditions and the minimal inhibitory concentration (MIC) defined 24–48 h later as described in table 1. Tests were done in triplicates and the table shows the average value of multiple MIC tests.

### Cytotoxicity assays of *Talaromyces wortmannii* extract and compounds in human cells

**Figure 2:**
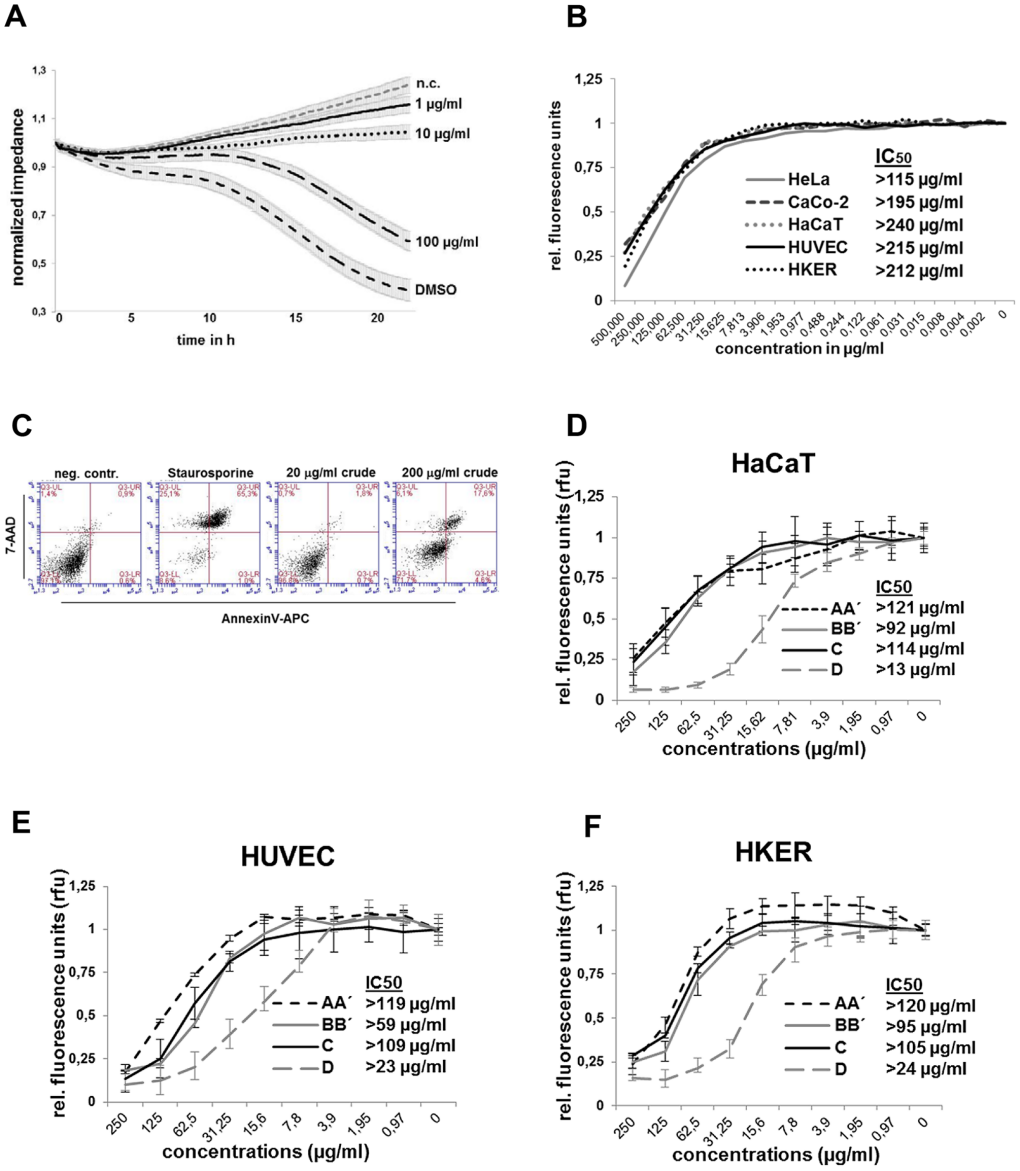
Cytotoxicity of *T. wormannii* extract and isolates. (A) HUVECs were plated at 10^6^ cells/ml on goldfilm electrods in 96 well plates and allowed to attach and form a monolayer for 24 h before cells were treated with 1, 10 or 100 µg/ml crude extract or 20% DMSO (control) or left untreated and cell toxicity measured every 5 min for 24 h. (B) HeLa, CaCo-2, HaCaT, HUVEC and HKER cells were plated at 2×10^5^ cells/ml in 96 well plates for 6 h and stimulated with 500 µg/ml–0.002 µg/ml crude extract for 24 h before Alamar blue was added (10%) and fluorescence intensity measured and the IC_50_ determined. (C) HUVECs were treated with 20 µg/ml or 200 µg/ml crude extract, with staurosporine (positive control) or left untreated (neg. control) for 24 h and apoptotic and necrotic cells measured using AnnexinV and 7-AAD. HaCaT (D), HUVEC (E) and HKER (F) were plated as described in B and cells stimulated with AA, BB, C and D.

In order to investigate the cytotoxic potential of *T. wortmannii* extract and compounds in detail, a number of experiments e.g. as ECIS measurement, Alamar Blue assays and AnnexinV/7AAD were performed. To estimate the proliferative or cytotoxic potential of the extract in real-time, Electrical Cell-Substrate Impedance Sensing (ECIS) technique was used as an accurate, automated and time-resolving method (Figure 2A). The cytotoxic potential can be monitored on-line, by means of measuring the decrease in impedance over time. As such, substances that have proliferative or cytotoxic potential can be detected, since they will lead to a changed resistance of the monolayer. HUVECs were cultured onto ECIS arrays and formed a confluent monolayer within 24 h (Figure 2A). The monolayers were treated with 1, 10 and 100 µg/ml *T. wortmannii* extract, with DMSO (control), or were left untreated before cytotoxic potential was subsequently assessed by continuous resistance measurements. Results demonstrated that the *T. wortmannii* extract increased the EC-monolayer disruption only at high concentrations (100 µg/ml) compared to the controls (Figure 2A). To determine the IC_50_ of *T. wortmannii* extract on primary endothelial cells (HUVEC), primary keratinocytes (HKER) and three different cell lines (HeLa, CaCo-2, HaCaT) 2×10^5^ cells per well in 96 well plates were allowed to attach for 6 h and cells incubated with *T. wortmannii* for 24 h before the Alamar Blue assay was performed (Figure 2 B). The IC_50_ value for all cell lines was >115 µg/ml, for the primary endothelial cell (HUVEC) >215 µg/ml and for the primary keratinocytes (HKER) >212 µg/ml, indicating that the extract only interfered with growth and viability at high concentrations. In order to distinguish between healthy, apoptotic, and necrotic cells, HUVEC cells were left untreated (neg. control) or were treated with 20 µg/ml or 200 µg/ml *T. wortmannii* extract or with staurosporin (pos. control) for 24 h before cells were stained with AnnexinV/7-AAD and assayed for apoptotic and necrotic characteristics that could be differentiated by FACS analysis (Figure 2 C). Compared to the negative control, only high concentrations induced apoptosis, but not necrosis.

In order to examine the cytotoxic potential of components AA, BB, C and D from *T. wortmannii*, HaCaT cells and the primary HUVEC and HKER were treated with different compound concentrations (250–0.97 µg/ml) for 24 h. The Alamar Blue assay was then performed and the IC_50_ values determined. As indicated in figures 2 D, E and F, compounds AA (IC_50_ >121 µg/ml; IC_50_>119 µg/ml_;_ IC_50_>120 µg/ml), BB (IC_50_ >92 µg/ml; IC_50_>59 µg/ml; IC_50_>95 µg/ml) and C (IC_50_>114 µg/ml; IC_50_>109 µg/ml; IC_50_>105 µg/ml) showed a very low cytotoxicity in all tested cells, whereas compound D demonstrated toxicity even at low concentrations in HaCaT (IC_50_ >13 µg/ml), HUVECs (IC_50_>23 µg/ml) and HKER cells (IC_50_>24 µg/ml).

### Effect of *Talaromyces wortmannii* extract and components on TNF-α induced NF-κB activation and ICAM-1 expression

**Figure 3:**
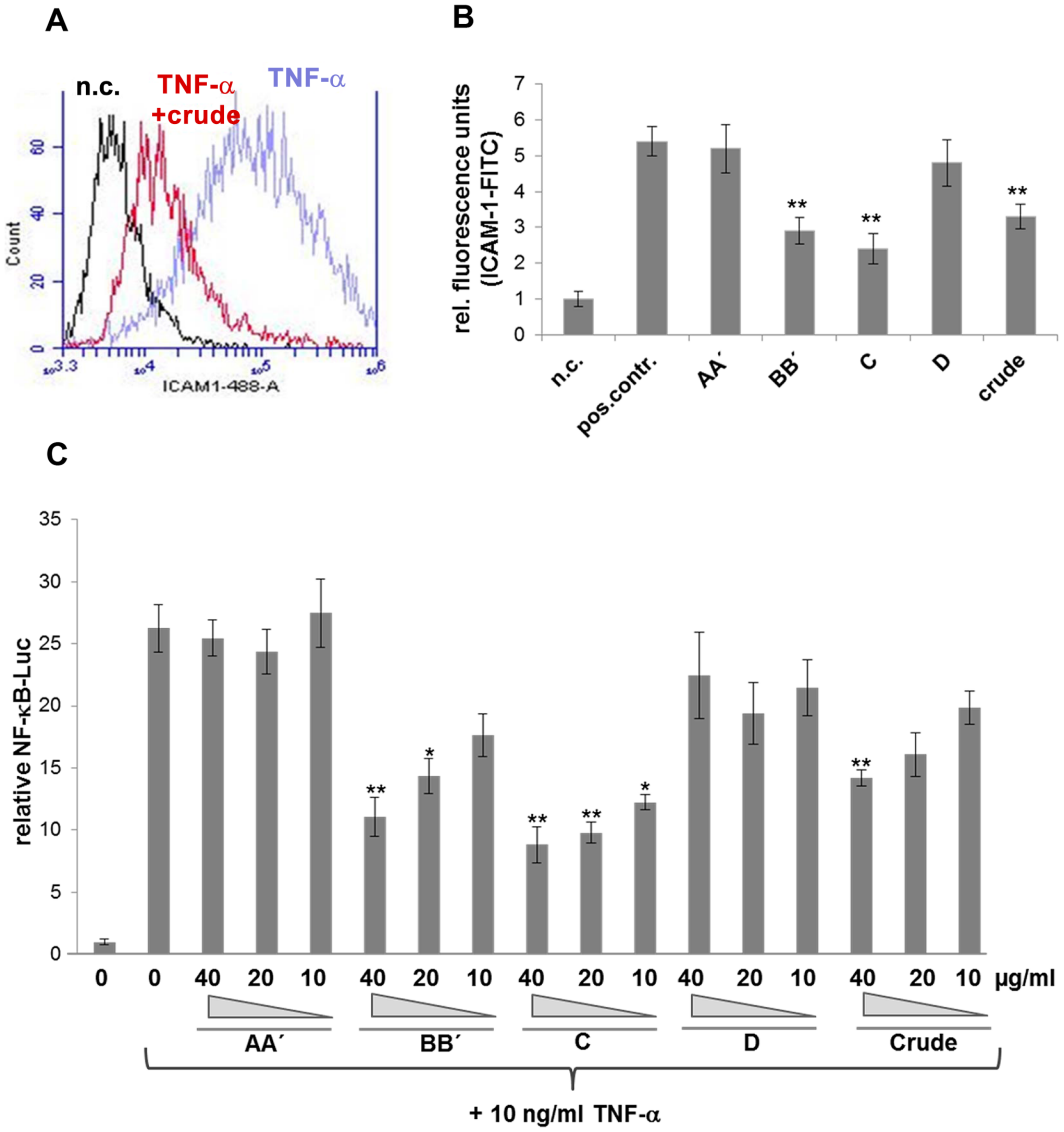
Screening for anti-inflammatory *T. wortmannii* components. (A) Flow cytometric analysis of ICAM-1 expression on the cell surface of HUVECs. Cells were stimulated with 10 ng/ml hTNF-α with or without 25 µg/ml crude extract or left untreated as negative control and stained with mouse anti-human ICAM-1 mAB. (B) Screening for anti-inflammatory substances, using a cell-based ELISA. Cells were treated for 1h in the absence or presence of 25 µg/ml substances AA, BB, C, D and crude extract, prior to being stimulated for 24 h with 10 ng/ml hTNF-α and stained with human ICAM-1 mAB (C) HEK293 cells were transiently transfected with NF-κB-Luciferase construct and EGFP construct. 24 h later, transfected cells were treated with 0, 40, 20 or 10 µg/ml substances or crude extract, prior to being stimulated with 10 ng/ml hTNF-α. Luciferase activities was determined 8 h later, normalized to the EFGP activities and expressed as fold increase over the control. (*, p<0.05; **, p<0.01, Students *t* test).

The following study was performed in a non-toxic range of the *T. wortmannii* extract and components (<40 µg/ml). We first investigated the effect of *T. wortmannii* extract on TNF-α-induced ICAM-1 expression in HUVEC cells. HUVECs were treated with 25 µg/ml crude extract for 1 h, and stimulated with TNF-α, and then ICAM-1 expression was measured by flow cytometry. As depicted in Fig. 3 A, the crude extract from *T. wortmannii* suppressed TNF-α-induced ICAM-1 expression. To investigate which of the substances was able to suppress ICAM-1 expression, HUVECs were plated in 96 well plates and treated with 25 µg/ml substance AA, BB, C, D and crude extract for 1 h, and stimulated further with TNF-α for 24 h and ICAM-1 expression measured as described in Material and Methods (Cell-based ELISA). As shown in Fig. 3 B, substances BB and C significantly suppressed TNF-α-induced ICAM-1 expression whereas AA and D showed no effect. To examine whether the substances BB and C were able to down regulate the TNF-α induced ICAM-1 expression via the inhibition of NF-κB activation, we evaluated the effect of the compounds on NF-κB promoter activity. HEK 293 cells transfected with an NF-κB promoter-luciferase construct were stimulated with TNF-α and luciferase activity measured. As depicted in Fig. 3C, substances BB and C and the crude extract inhibited NF-κB promoter activity in a dose-dependent manner. These results propose that the crude extract and the substances BB and C from *T. wortmannii* have inhibitory activities on the signalling pathways that lead to activation of NF-κB and the resulting ICAM-1 expression.

### Effect of *Talaromyces wortmannii* component C on NF-κB and MAPK activation induced by *Propionibacterium acnes*

**Figure 4:**
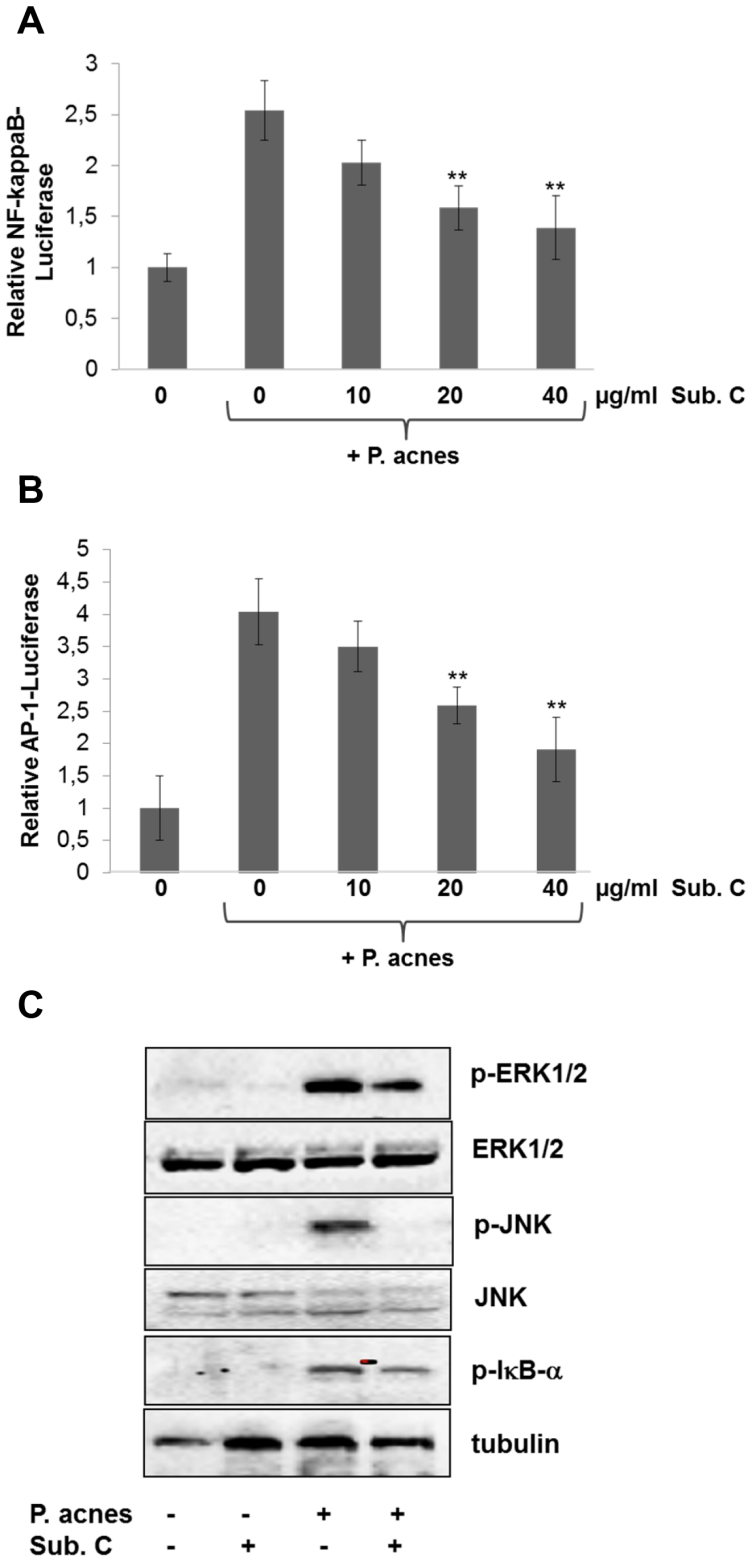
Effect of *T. wortmannii* substance C on *P. acnes*-induced activation of NF- κ**B and AP1.** HaCaT cells were transiently transfected with NF-κB- (A) or AP-1- Luciferase (B) as described in Fig. 3C and 24 h later treated with 0, 10, 20 or 40 µg/ml component C. After 2 h cells were then stimulated by incubation with *P. acnes* suspension and luciferase activities measured 8 h later. The data are the mean of three experiments +− standard deviation. (C) HaCaT cells were pre-treated with component C (Sub. C) for 2 h, and then stimulated with *P. acnes* suspension for 20 min. Phosphorylation and total protein expression of ERK, JNK, and IkB-α were detected by western blot analysis using specific antibodies. Tubulin was used as a loading control.

Since component C of all isolated and tested substances was found to show low toxicity, potent anti-inflammatory activity and high antibacterial capacity especially against *P. acnes* (Table 2, Fig. 2D, 3 B, C), we therefore concentrated on this compound and its capability to suppress *P. acnes* induced inflammation. There is ample evidence that NF-κB and AP-1 play an important role in inflammation [20]. Therefore, we transfected HaCaT cells with NF-κB and AP-1 reporter plasmids respectively and examined the effect of component C on *P. acnes*-induced NF-κB and AP-1 expression. As shown in Fig. 4 A and B, component C treatment resulted in down regulation of both NF-κB and AP-1 in a concentration dependent manner. To verify these results, HaCaT cells were treated with component C and inflammation stimulated with *P. acnes* for 20 min. Component C abolished *P. acnes*-induced phosphorylation of the NF-κB-Inhibitor κB-alpha, and the MAP kinases ERK and JNK (Fig. 4C).

### Influence of *Talaromyces wortmannii* components on production of pro-inflammatory chemokine IL-8 induced by *Propionibacterium acnes*

**Figure 5:**
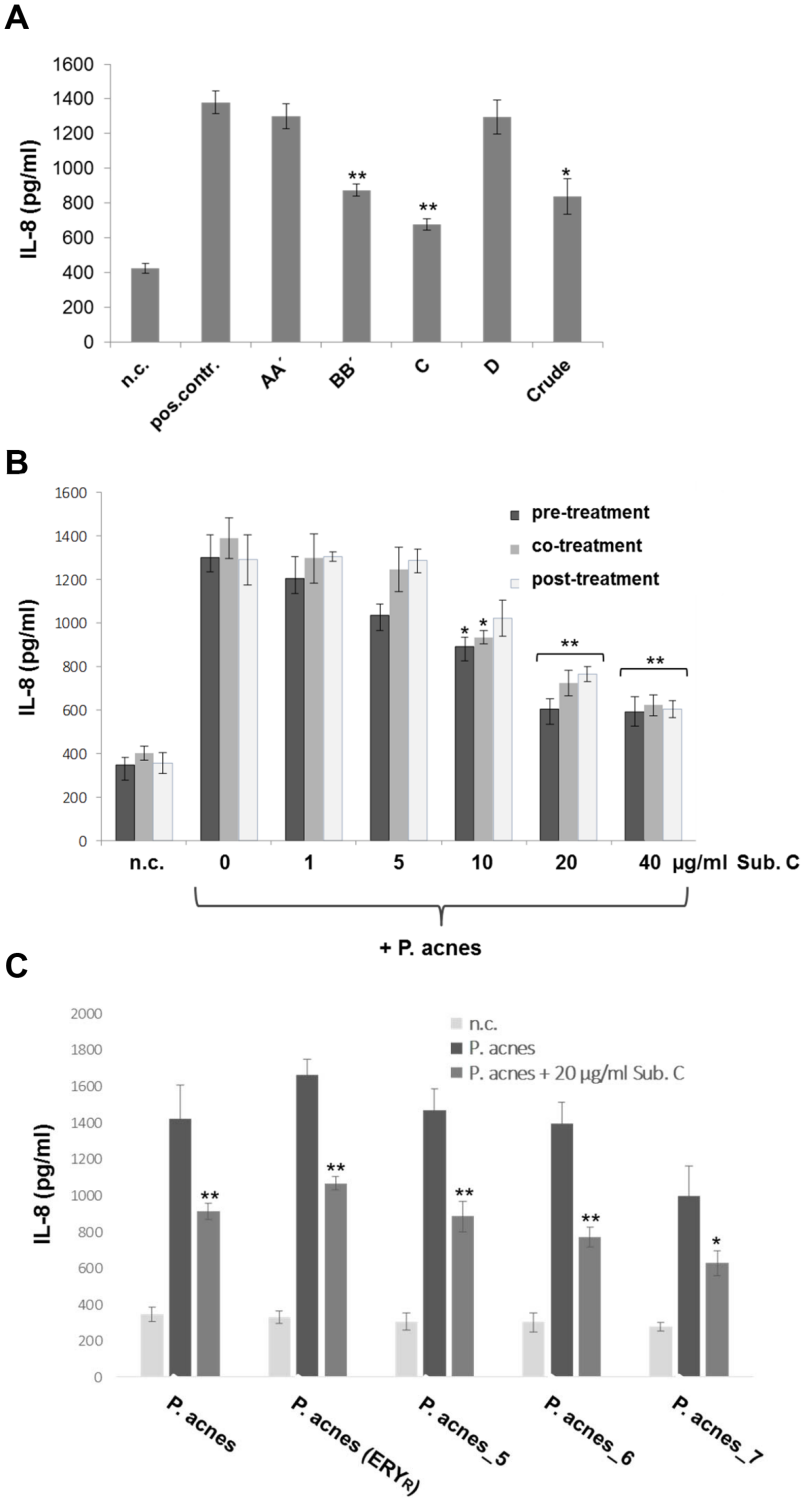
Effect of *T. wortmannii* components on *P. acnes*-induced IL-8 expression. (A) HaCaT cells were pre-treated with 20 µg/ml components AA, BB, C, D, Crude extract or left untreated for 2 h and IL-8 production stimulated with *P. acnes* suspension for 24 h. Control experiments were run with cells alone (n.c.) or cells stimulated with *P. acnes* suspension (pos. contr.). (B) Pre-, co-, and post-treatment effects of several concentrations of component C on *P. acnes*–induced IL-8 expression in HaCaT cells. (C) HaCaT cells were stimulated with different *P. acnes* strains (*P. acnes*, *P. acnes* (ERY^R^), *P. acnes*_5-7) and post-treated with 20 µg/ml component C 3 h later. Data are presented as the mean+- standard deviation of three independent experiments. Statistical significance is indicated by *, p<0.05; **, p<0.01, Students *t* test.

Having shown that *P. acnes*-induced NF-κB and AP-1 expression is inhibited by compound C, we next investigated the production of the target protein IL-8, a potent chemoattractant for neutrophils, in keratinocytes stimulated with *P. acnes* in the presence of *T. wortmannii* components and extract. We accomplished this test by pre-incubation of HaCaT cells with components AA, BB, C, D and crude extract and stimulated with *P. acnes* for 24 h whilst monitoring for the production of the pro-inflammatory chemokine IL-8. Components BB and C as well as the crude extract significantly decreased IL-8 production, whereas components AA and D showed no effect (Fig. 5A). We next analysed the influence of component C on IL-8 production, by stimulating HaCaT cells with component C (1–40 µg/ml) and *P. acnes*. Component C decreased IL-8 production when administered as pre-, co- and post-treatment in a dose-dependent manner (Fig. 5B). Post-treatment with 20 µg/ml of component C significantly (p<0.01 for *P. acnes*, *P. acnes* (ERY^R^), *P. acnes*_5 and 6; p<0.05 for *P. acnes*_7) reduced *P. acnes*-induced IL-8 production after 24 h incubation (Fig. 5C). These results are consistent with those obtained for the inhibition of NF-κB and AP-1. These data suggest that the inhibition by *T. wortmannii* component BB and C or extract of *P. acnes*-induced IL-8 production in HaCaT cells is caused by down regulation of the NF-κB and MAPK pathway.

## Discussion

Based on the fact that endophytes are generally described to be a potential source of promising bioactive products and that the secondary metabolites of the endophyte *T. wortmannii* from *Aloe vera* have previously demonstrated antibacterial activity against multi resistant *Staphylococcus aureus* [17], [21], [22], we first screened the EtOAc extract from *T. wortmannii* (Figure 1 A) for their antibacterial and cytotoxic (Figure 2 A-C) activities. Interestingly, the crude extract indicated a high minimal inhibitory concentration (MIC) activity against the Gram-positive strains tested (3.8–31.5 µg/ml) but only low activity against Gram-negative bacteria (>62.8 µg/ml). These data are confirmed previously by the Group of Peter Proksch who reported on new antibacterial agents produced from *T. wortmannii* which retain full activity against clinical isolates of multi-drug resistant bacteria [17]. The current work indicated that the EtOAc extract from *T. wortmannii* contained potential antimicrobial activity against *MRSA* (15.7 µg/ml), *E. faecalis* (7.8 µg/ml), *S. epidermidis* (7.8 µg/ml) but the highest activity was observed against *P. acnes* (3.9 µg/ml; Table 1). In addition, our results also indicated that the *in vitro* toxicity of the EtOAc extract from *T. wortmannii* was very low. Using the Electrical Cell Substrate Impedance Sensing (ECIS) method, we clearly demonstrated in real-time that the endothelial monolayer (HUVECs) was disrupted only when the crude extract was applied in high concentrations (100 µg/ml; Figure 2 A). To evaluate the cytotoxicity in more detail, cell metabolism was measured and the IC_50_ determined (Figure 2 B). The EtOAc extract from *T. wortmannii* showed cytotoxicity only in very high concentrations (IC_50_: HeLa >115 µg/ml; CaCo-2 >195 µg/ml; HaCaT >240 µg/ml; HUVEC >215 µg/ml and HKER >212 µg/ml) suggesting a specific antibacterial activity against Gram-positive bacteria and a therapeutic window of more than 200 µg/ml by comparing the MIC against *P. acnes* and cytotoxicity performed with primary keratinocytes (HKER), primary endothelial cells (HUVEC) or HaCaT cells.

Using an isolation and purification method as described previously [17], [23]–[25], six different molecules namely AA, BB, C and D could be detected in the course of this study in the HPLC-UV/Vis (260 nm) profile (Figure 1 A). These compounds were isolated as described in Materials and Methods (Figure 1 B). In comparison, Bara et al. (2013) purified seven compounds from the endophytic fungus *T. wortmannii isolated* from *Aloe vera*. Compounds 1 and 2 were identified as flavomannin or atropodiastereomer of flavomannin, respectively, resembling our compounds A and A. Compounds 3 and 4 were identified as new natural heterodimers of flavomannin and resembled our compounds B and B with an m/z ratio of 561 [M+H^+^], and were also found to represent a pair of atropodiastereomers. A third part of compounds, 5 and 6 published by Bara et al. (2013) was revealed to have a molecular weight of 542 g/mol and identified as novel products named Talaromannin A and B. These new molecules are heterodimers of an atrochrysone moiety with an emodin residue, and compound 6 was indicated to be an atropodiastereomer of compound 5. Remarkably- and in contrast to the other two compound pairs, AA' and BB'- we could identify only one molecule with an m/z ratio of 543 [M+H^+^], designated as compound C. The lack of a stereochemical congener of our single compound C might be due to its immanent stereochemical characteristics, being different from those inherent to both Talaromannins A and B, potentially produced by diverging configurations at the chiral centers C-3/3′. However, this point remains unclear, since the configurations at the carbon centers C-3/3′ remain undefined for the two Talaromannins A and B (Bara et al. (2013)). Compound D, which was the most apolar molecule in the mixture was isolated and identified as the known anthraquinone skyrin with a molecular weight of 538.46 g/mol [17]. Bara et al. (2013) clearly demonstrated that all six compounds exhibited antibacterial activity against staphylococci, including against high-level (multi) drug-resistant isolates, with MIC values from 4–8 µg/ml for the most active compounds. We demonstrated that all 5 compounds displayed antibacterial activity, but the highest activity was obtained from compound C with a MIC of 0.49–1.98 µg/ml for *P. acnes,* including erythromycin-resistant isolates, 0.24 µg/ml for *S. epidermidis*, and 1.9 µg/ml for *MRSA* (Table 2 and 3). All the other isolated compounds (compound AA', BB' and D) demonstrated antibacterial activity against the tested isolates with MIC values of 4 to 62.5 µg/ml observed. Furthermore, upon closer examination, it was revealed that component C demonstrated bacteriostatic activity at low concentrations and lytic activity at concentrations higher than >20 µg/ml when tested against *P. acnes* (data not shown).

Interestingly, except component D (IC_50_ of >13 µg/ml for HaCaT, >23 µg/ml for HUVECs and >24 µg/ml for HKER), components AA, B and C presented cytotoxicity only in very high concentrations with an IC_50_ of >92 µg/ml for HaCaT cells, >59 µg/ml for HUVECs and >95 µg/ml for HKER cells. *In vivo* toxicity studies with component C, where guinea pigs were topically treated twice a day for 5 days with a 2% cream (1% DMSO in Ultrabas) showed no toxic effects or skin irritations compared to control (formulation without component C; data not shown). Comparing the MIC values for the different substances with the *in vitro* cytotoxicity (IC_50_) data, it was shown that the highest antibacterial activity was found in component C, especially against *P. acnes,* with a therapeutic window of more than 113 µg/ml for HaCaT cells, >108 µg/ml for HUVECs, and >105 µg/ml for HKER.

There have been several reports that antibacterial substances have anti-inflammatory activity [26], [27], [28]. Since the EtOAc extract was shown to downregulate TNF-induced ICAM-1 upregulation in HUVECs (Figure 3 A), we further characterized compounds AA, B, C and D for their anti-inflammatory potential using a cell-based ICAM-1 ELISA [19]. Both compounds BB and C proved to be anti-inflammatory substances with an inhibitory activity of 46% for BB or more than 55% for compound C, whereas AA and D had no significant anti-inflammatory activity. Expression of several pro-inflammatory cytokines and intercellular adhesion molecules such as ICAM-1 have been reported to be regulated by NF-κB activation in a wide variety of cells [29], [30]. In this study, we found that compounds BB and C inhibit TNF-induced NF-κB activation in a concentration dependent manner. However, the mechanism by which these compounds inhibit NF-κB activation remains unclear. Since compound C or the EtOAc extract were able to inhibit *P. acnes* at very low concentrations and also were able to downregulate TNF-induced ICAM-1 upregulation in human endothelial cells via the NF-κB pathway, we used further *in vitro* models of inflammation to characterise whether these compounds might prove to be therapeutically interesting for the treatment of acne. The induction of inflammatory mediators such as IL-8 in response to *P. acnes* can be regulated by the activation of transcription factors, including NF-κB and AP-1 in various cell types [13], [31], [32]. IL-8 is a chemokine released during the inflammatory stage of acne and is important for the activation and chemoattraction of immune and inflammatory cells [33], [34]. In this study, we have shown that all tested *P. acnes* strains induced an increase in IL-8 release in human keratinocytes with a corresponding increase in the activation of NF-κB and AP-1. IL-8 release was inhibited by the EtOAc extract, compounds BB and C. Compound C proved to be the molecule which exhibited the most effective IL-8 inhibition after *P. acnes* stimulation (20 µg/ml causes >50% IL-8 inhibition) in HaCaT cells (Figure 5 A), suggesting that this compound may interfere with the transcription factors NF-κB and/or AP-1, required for the transcription of the IL-8 gene. Both NF-κB and AP-1 transactivation induced by *P. acnes* were inhibited by compound C in a concentration dependent manner (Figure 4 A and B). We further specified that compound C inhibited the activation of the MAP kinases p-ERK1/2 and p-JNK and the inhibitor of kappa B (Figure 4 C). This strongly suggests that the EtOAc extract from *T. wortmannii* and, in particular compound C, have anti-inflammatory properties and may have the potential to attenuate inflammation stimulated by *P. acnes*. In addition, compound C significantly reversed the inflammatory effect when administered at the same time as *P. acnes* (co-treatment), or at a later time point (post-treatment) in all tested isolates, including erythromycin-resistant mutants (Figure 5 B and C). Since the incidence of antibiotic resistance in acne has continued to rise over the recent decades [36], antibiotics with antimicrobial and anti-inflammatory properties such as compound C are promising treatments for acne vulgaris.

## Conclusions

In summary, this study suggests that the compounds isolated from *T. wortmannii* demonstrate a reasonable mechanism of activity against acne. We show here the isolation of the different compounds from *T. wortmannii* and reveal that compound C in particular has strong antibacterial activity especially against *P. acnes*, demonstrates low cytotoxicity and has anti-inflammatory properties. Furthermore, the anti-inflammatory properties of compound C were attributable to its ability to inhibit IL-8 release by blocking NF-κB and AP-1 activation. Therefore, compound C has promising characteristics to be used as a potential antibacterial/anti-inflammatory molecule for the treatment of acne vulgaris.
